# Integration of Lipidomics and Transcriptomics Reveals Reprogramming of the Lipid Metabolism and Composition in Clear Cell Renal Cell Carcinoma

**DOI:** 10.3390/metabo10120509

**Published:** 2020-12-13

**Authors:** Giuseppe Lucarelli, Matteo Ferro, Davide Loizzo, Cristina Bianchi, Daniela Terracciano, Francesco Cantiello, Lauren N. Bell, Stefano Battaglia, Camillo Porta, Angela Gernone, Roberto A. Perego, Eugenio Maiorano, Ottavio de Cobelli, Giuseppe Castellano, Leonardo Vincenti, Pasquale Ditonno, Michele Battaglia

**Affiliations:** 1 Department of Emergency and Organ Transplantation-Urology, Andrology and Kidney Transplantation Unit, University of Bari, 70124 Bari, Italy; d.loizzo27@gmail.com (D.L.); pasquale.ditonno@uniba.it (P.D.); michele.battaglia@uniba.it (M.B.); 2 Division of Urology, European Institute of Oncology (IEO)-IRCCS, 20141 Milan, Italy; matteo.ferro@ieo.it (M.F.); ottavio.decobelli@ieo.it (O.d.C.); 3 School of Medicine and Surgery, University of Milano-Bicocca, 20126 Monza, Italy; cristina.bianchi@unimib.it (C.B.); roberto.perego@unimib.it (R.A.P.); 4 Department of Translational Medical Sciences, University of Naples “Federico II”, 80138 Naples, Italy; daniela.terracciano@unina.it; 5 Department of Urology, Magna Graecia University of Catanzaro, 88100 Catanzaro, Italy; cantiello@unicz.it; 6 Metabolon, Inc., Research Triangle Park, Morrisville, NC 27519, USA; lbell@metabolon.com; 7 Department of Interdisciplinary Medicine, University of Bari, 70124 Bari, Italy; battaglias@hotmail.it; 8 Department of Biomedical Sciences and Clinical Oncology (DIMO), Medical Oncology Unit, University of Bari, 70124 Bari, Italy; camillo.porta@gmail.com (C.P.); angelagernone@libero.it (A.G.); 9 Department of Emergency and Organ Transplantation-Pathology Unit, University of Bari, 70124 Bari, Italy; eugenio.maiorano@uniba.it; 10 Department of Medical and Surgical Sciences, Nephrology Dialysis and Transplantation Unit, University of Foggia, 71122 Foggia, Italy; castellanogiuseppe74@gmail.com; 11 Division of General Surgery, Polyclinic Hospital, 70124 Bari, Italy; leonardovincenti@gmail.com; 12 Department of Urology, National Cancer Institute “Giovanni Paolo II”, 70124 Bari, Italy

**Keywords:** renal cell carcinoma, lipidomics, SCD1, cholesterol, lipids

## Abstract

Clear cell renal cell carcinoma (ccRCC) is fundamentally a metabolic disease. Given the importance of lipids in many cellular processes, in this study we delineated a lipidomic profile of human ccRCC and integrated it with transcriptomic data to connect the variations in cancer lipid metabolism with gene expression changes. Untargeted lipidomic analysis was performed on 20 ccRCC and 20 paired normal tissues, using LC-MS and GC-MS. Different lipid classes were altered in cancer compared to normal tissue. Among the long chain fatty acids (LCFAs), significant accumulations of polyunsaturated fatty acids (PUFAs) were found. Integrated lipidomic and transcriptomic analysis showed that fatty acid desaturation and elongation pathways were enriched in neoplastic tissue. Consistent with these findings, we observed increased expression of stearoyl-CoA desaturase (SCD1) and FA elongase 2 and 5 in ccRCC. Primary renal cancer cells treated with a small molecule SCD1 inhibitor (A939572) proliferated at a slower rate than untreated cancer cells. In addition, after cisplatin treatment, the death rate of tumor cells treated with A939572 was significantly greater than that of untreated cancer cells. In conclusion, our findings delineate a ccRCC lipidomic signature and showed that SCD1 inhibition significantly reduced cancer cell proliferation and increased cisplatin sensitivity, suggesting that this pathway can be involved in ccRCC chemotherapy resistance.

## 1. Introduction

Renal cell carcinoma (RCC) accounts for about 2–3% of all malignant diseases in adults. GLOBOCAN 2018 estimates of worldwide cancer incidence and mortality were 403,262 new cases and 175,098 deaths for kidney cancer [1]. Recent estimates have calculated that, in 2020, in the United States, 73,750 new cases will be diagnosed and 14,830 patients will die of this tumor [2]. The rediscovery of cancer as a metabolic disorder has led to the identification of specific oncometabolites with an important role in tumor growth and progression [3,4,5,6]. The introduction of high-throughput omics technologies has led not only to a detailed molecular characterization of RCC, but also to the identification of biomarkers that allow a more accurate prognostic stratification [7]. The discovery of novel markers will play an important role in the clinical management of this disease considering that up to 30% of cases have a metastatic disease at diagnosis and that, to date, we have no specific molecular factor for diagnosis and prognostic stratification [8,9,10,11]. Recent studies have shown that RCC is fundamentally a metabolic disease, since many genes that are altered in this tumor play a fundamental role in controlling cell metabolic activities [12,13,14]. Indeed, we showed that, in clear cell renal cell carcinoma (ccRCC), a metabolic reprogramming occurs, involving the glucose metabolism and the pentose phosphate pathway, and that patients with high levels of glycolytic enzymes had reduced survival rates [15,16,17]. In accordance with these findings, the role of NADH dehydrogenase 1 alpha subcomplex 4-like 2 (NDUFA4L2) in controlling ccRCC bioenergetics and other cancer cell activities such as proliferation, migration, mitophagy, angiogenesis, and chemotherapy resistance, was demonstrated [18]. Moreover, additional data indicate that glucose and lipid metabolism reprogramming is grade-dependent, suggesting the need for a ccRCC reclassification on the basis of these particular metabolic alterations [19]. Given the importance of metabolic reprogramming in cancer cells, and the involvement of lipids in many cellular processes such as membrane remodeling and cell signaling, we delineated a lipidomic profile of human ccRCC, and integrated it with transcriptomic data to connect the variations in cancer lipid metabolism with gene expression changes.

## 2. Results

### 2.1. Lipidomic Profile Distinguishes ccRCC from Normal Renal Tissue

**Figure 1:**
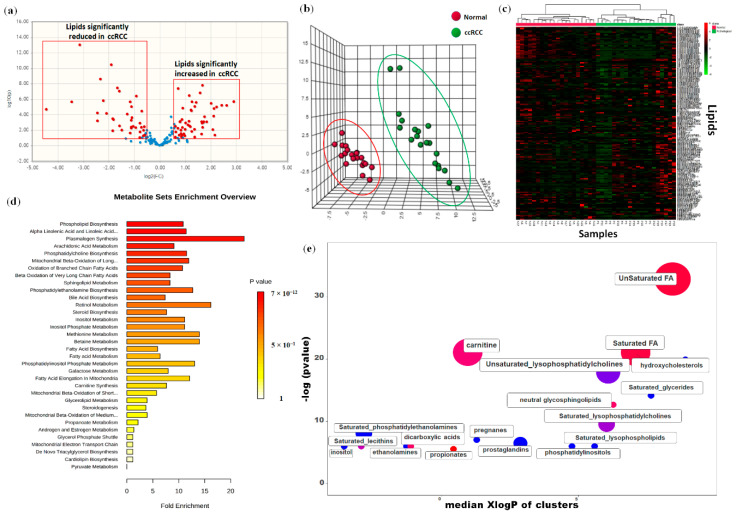
Volcano plot of the 158 lipids profiled (**a**). Principal component analysis (PCA) of the global tissue lipidome demonstrated that the two groups (clear cell renal cell carcinoma (ccRCC) vs. normal renal tissue) were clearly distinguishable (**b**). Hierarchical clustering heatmap analysis of lipids in normal and cancer tissues (**c**). Metabolic set enrichment analysis (MSEA) showing the most altered biochemical metabolic pathways in ccRCC (**d**). ChemRICH set enrichment statistical plot. Each node reflects a significantly altered cluster of lipids. Node sizes represent the total number of lipids in each cluster set. The node color scale shows the proportion of increased (red) or decreased (blue) compounds in tumor compared to normal tissue. Purple nodes have both increased and decreased lipids (**e**).

Untargeted lipidomic analysis was performed on 40 kidney-derived tissues, including 20 ccRCC and 20 paired normal tissues, using LC-MS and GC-MS platforms. In total, 158 lipids were identified, and 93 were found to be differentially expressed in tumor tissues compared to normal samples (57 higher and 36 lower) (Figure 1a). The application of principal component analysis (PCA) to distinguish normal and pathological samples as a function of the global tissue lipidome demonstrated that the two groups were clearly different (Figure 1b). In accordance with PCA, hierarchical clustering analysis and heatmap visualization showed a clear distinction between ccRCC and non-neoplastic tissue (Figure 1c).

To obtain a global overview of altered biochemical processes, we performed a metabolite set enrichment analysis (MSEA) using MetaboAnalyst 4.0 (https://www.metaboanalyst.ca) [20], and an alternative enrichment analysis based on chemical similarity (ChemRICH) (https://chemrich.fiehnlab.ucdavis.edu) [21]. These functional approaches showed that alterations in glycerophospholipid metabolism, in arachidonic acid and prostaglandin production, in biosynthesis of unsaturated fatty acid and fatty acid elongation, had the highest impact on the ccRCC lipidome (Figure 1d,e).

### 2.2. Global Lipidomic Profile of ccRCC

**Figure 2:**
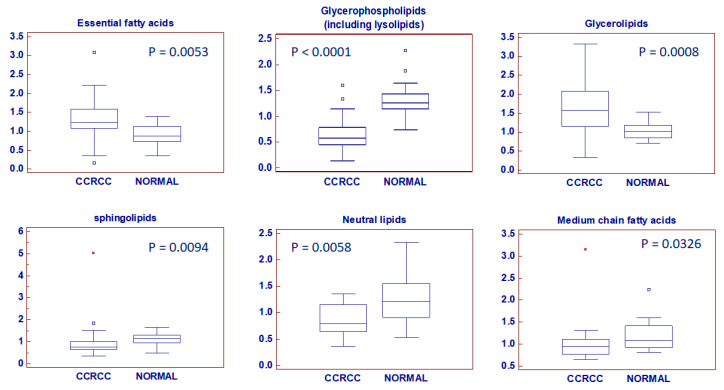
Lipid classes differentially accumulated between neoplastic (ccRCC) and normal tissue. Y-axis: metabolite relative amount. Small squares indicate outlier in box-and-whisker plots. Solid square indicates extreme outlier.

Different lipid classes were identified. These included essential fatty acids (EFAs), glycerophospholipids (including lyso species), glycerolipids, sphingolipids, neutral lipids (including sterols/steroids), medium chain fatty acids (MCFAs), long chain fatty acids (LCFAs), eicosanoids, and carnitine metabolism-related intermediates. In particular, in cancer tissue we found significantly higher levels of EFAs (*p* = 0.005), glycerolipids (*p* = 0.0008), LCFAs (*p* = 0.001), and carnitine-related metabolites (*p* = 0.01). The other lipid classes were higher in normal tissue compared to ccRCC (Figure 2).

**Figure 3:**
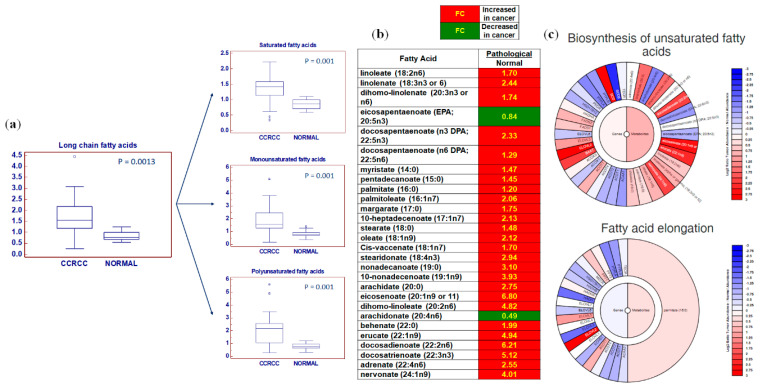
Among the long chain fatty acids (LCFAs), saturated fatty acids (SFAs), monounsaturated fatty acid (MUFAs), and polyunsaturated fatty acids (PUFAs) were significantly accumulated in cancer (ccRCC) compared to normal tissue (**a**). Schematic model summarizing the differences in free fatty acids between normal and tumor tissue (**b**). Exploration of the “Metabologram” Data Portal. The changes in the “Biosynthesis of unsaturated fatty acids” and “Fatty acid elongation” pathways are shown in both transcripts and metabolites when comparing tumors to adjacent normal kidney tissues (**c**). Y-axis: metabolite relative amount. Small squares indicate outlier in box-and-whisker plots.

Among the LCFAs, significant accumulations of saturated fatty acids (SFAs), monounsaturated fatty acid (MUFAs), and polyunsaturated fatty acids (PUFAs) were found (all *p* = 0.001, Figure 3a–c).

**Figure 4:**
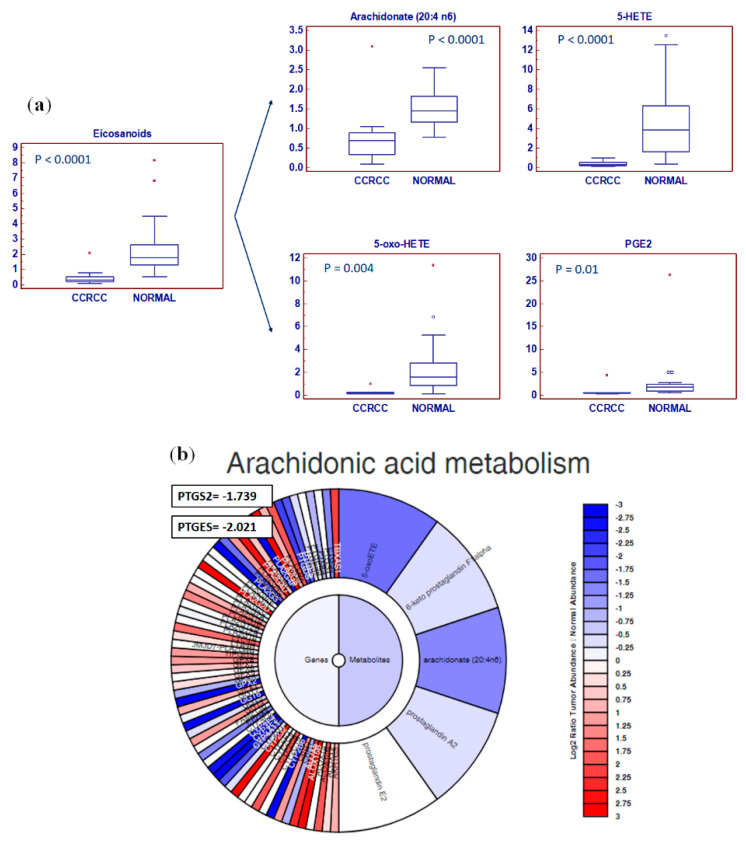
The tissue levels of eicosanoids were reduced in tumor samples (ccRCC) compared to normal kidney (**a**). Exploration of the “Metabologram” Data Portal. The changes in the “Arachidonic acid metabolism” pathway are shown in both transcripts and metabolites when comparing tumors to adjacent normal kidney tissues (**b**). Y-axis: metabolite relative amount. Small squares indicate outlier in box-and-whisker plots. Solid square indicates extreme outlier.

Interestingly, the tissue levels of eicosanoids were reduced in pathological samples. In particular, we found reduced amounts of arachidonate (*p* < 0.0001), 5-hydroxyeicosatetraenoic acid (5-HETE) (*p* < 0.0001), 5-oxo-eicosatetraenoic acid (5-oxo-ETE) (*p* = 0.004), and prostaglandin E2 (*p* = 0.01) (Figure 4).

**Figure 5:**
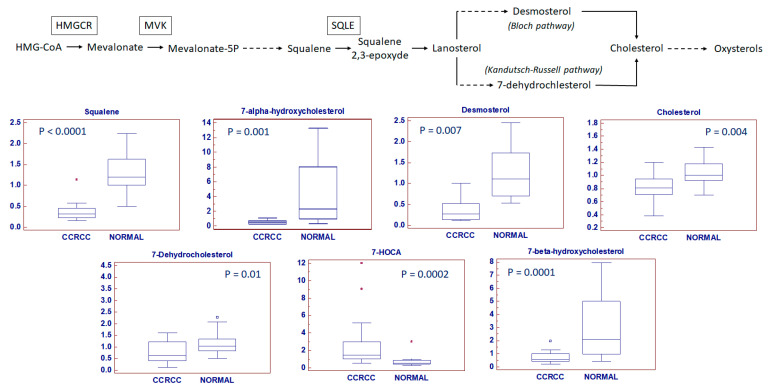
Cholesterol biosynthesis pathways. A reduced accumulation of main metabolic intermediates in both Kandutsch-Russell and Bloch pathways was observed in neoplastic tissue (ccRCC). Y-axis: metabolite relative amount. Small squares indicate outlier in box-and-whisker plots. Solid square indicates extreme outlier.

Cholesterol biosynthesis pathways were also analyzed. Clear cell RCC was characterized by a reduced accumulation of the main metabolic intermediates in both the Kandutsch–Russell and Bloch pathways (Figure 5). In addition, we found reduced levels of two oxysterols, namely 7-alpha-hydroxy- and 7-beta-hydroxy-cholesterol (*p* = 0.001 and *p* = 0.0001, respectively), and increased concentrations of 7α-Hydroxy-3-oxo-4-cholestenoic acid (7-HOCA) (*p* = 0.0002), a cholesterol-derived metabolite that is also increased in prostate cancer (Figure 5). Moreover, we found a positive correlation between serum and tissue levels of total cholesterol in the neoplastic samples (Rs = 0.98, *p* < 0.0001).

### 2.3. Integrated Lipidomic and Transcriptomic Analysis

**Figure 6:**
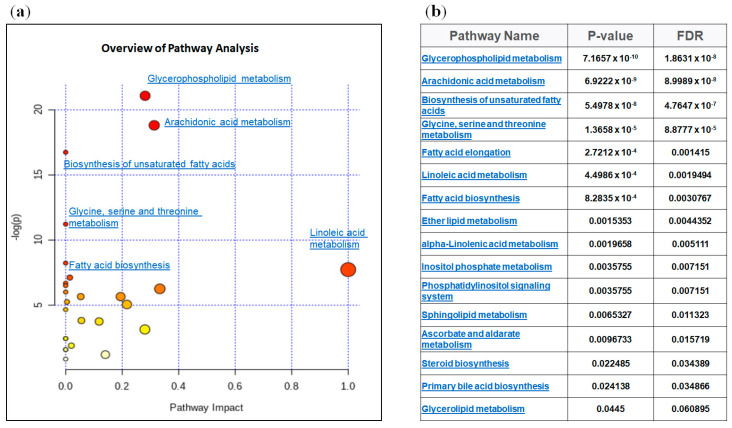
Integrated metabolic pathway enrichment analysis. All the matched pathways are displayed as circles. The color and size of each circle are based on the *p*-value and pathway impact value, respectively (**a**). Results of pathway analysis according to *p*-value and false discovery rate (FDR) (**b**).

To compare the relative changes in gene expression and lipid abundance in ccRCC, we integrated the lipidomic data with gene expression data from 10 ccRCC tumor samples and matched non-tumor kidney tissue samples obtained from patients who underwent nephrectomy in our department (GSE47032). The combined analysis identified significantly enriched biochemical pathways (*p* < 0.05), including those of unsaturated fatty acid biosynthesis, glycerolipid, glycerophospholipid and arachidonic acid metabolism (Figure 6a,b).

### 2.4. Clear Cell RCC Displays an Altered Expression Profile of Lipid Metabolism-Related Genes

**Figure 7:**
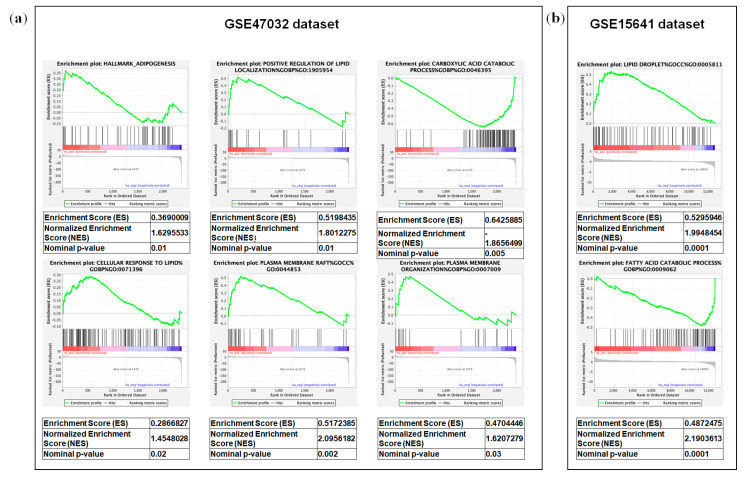
Gene set enrichment analysis (GSEA) of the GSE47032 (**a**) and GSE15641 dataset (**b**).

Gene set enrichment analysis (GSEA) [22] of the GSE47032 dataset showed that ccRCC featured multiple enriched gene sets depicting adipogenesis, cellular response to lipids, plasma membrane rafts, regulation of lipid localization, plasma membrane organization, and reduced catabolism of carboxylic acid (Figure 7a). To confirm the specific contribution of altered gene expression to the global lipidomic profile of ccRCC, we also performed GSEA in Jones cohort (GSE15641) including 23 normal kidney samples and 32 ccRCC (Figure 7b).

**Figure 8:**
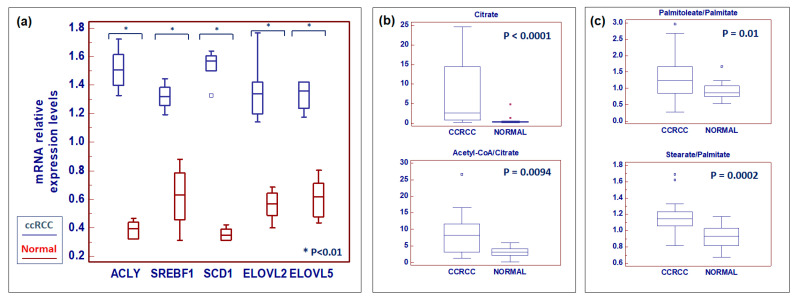
Analysis of gene expression by Real time PCR of ATP citrate lyase (ACLY), sterol regulatory element-binding transcription factor 1 (SREBF1), stearoyl-CoA desaturase-1 (SCD1) and fatty acid elongase 2 and 5 (ELOVL2 and ELOVL5) (**a**). Levels of citrate and acetyl-CoA-to-citrate ratio are increased in ccRCC compared to normal tissue (**b**). Palmitoleate-to-palmitate ratio and stearate-to-palmitate ratio are increased in ccRCC (**c**). Small squares indicate outlier in box-and-whisker plots. Solid square indicates extreme outlier.

Next, we evaluated the mRNA expression of four enzymes involved in fatty acid biosynthesis, desaturation and elongation, namely, ACLY, SREBF1, SCD1, and ELOVLs (Figure 8a). ATP citrate lyase (ACLY) generates acetyl-CoA from citrate, and it connects carbohydrate metabolism with fatty acid biosynthesis. We found an increased expression of ACLY in association with elevated levels of citrate and increased acetyl-CoA-to-citrate ratio in ccRCC compared to normal tissue (Figure 8b). Sterol regulatory element-binding transcription factor 1 (SREBF1), which encodes for a key transcriptional regulator of lipid metabolism, was also upregulated in tumor tissue (Figure 8a). Multi-omics analysis showed that fatty acid desaturation and elongation pathways were enriched in neoplastic tissue. Consistent with these findings, we observed an increased expression of stearoyl-CoA desaturase-1 (Δ-9-desaturase; SCD1) and fatty acid elongase 2 and 5 (ELOVL2 and ELOVL5) in ccRCC (Figure 8a). Next, we evaluated the palmitoleate-to-palmitate ratio and the stearate-to-palmitate ratio as readouts of SCD-dependent desaturation and ELOVL-dependent FA elongation pathways, respectively. We found that in cancer tissue, both ratios were significantly higher as a result of increased enzymatic activity (Figure 8c).

**Figure 9:**
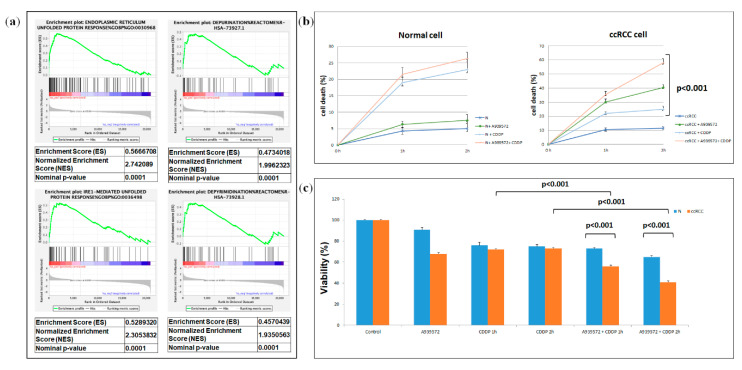
Gene set enrichment analysis (GSEA) of the GSE41485 dataset (**a**). SCD1 has a role in RCC resistance to cisplatin (CDDP)-induced cytotoxicity (C). The death rate of treated tumor cells (tumor + A939572 + CDDP) is significantly higher than that of untreated cells (tumor + CDDP) (*p* < 0.001). No difference is observed in normal cells (*p* > 0.05) (**b**). MTT assay reveals significantly decreased cell viability when RCC cells are treated with A939572 before cisplatin incubation (**c**).

GSEA of the GSE41485 dataset showed that SCD1 inhibition in ccRCC cells induced the activation of the unfolded protein response (UPR) and depurination/depyrimidination processes (Figure 9a). Therefore, we explored the role of SCD1 in sustaining cancer cell proliferation and in reducing cisplatin-induced cytotoxicity. Primary renal cancer cells treated with a small molecule SCD1 inhibitor, A939572, proliferated at a slower rate than non-treated cancer cells. In addition, after cisplatin treatment, the death rate of tumor cells treated with A939572 was significantly greater than that of untreated cancer cells (*p* < 0.001, Figure 9b). The 3-(4,5-dimethylthiazol-2-yl)-2,5 diphenyl tetrazolium bromide (MTT) assay confirmed these findings, demonstrating a decreased cell viability when tumor cells were treated with A939572 before cisplatin incubation (Figure 9c). 

**Figure 10:**
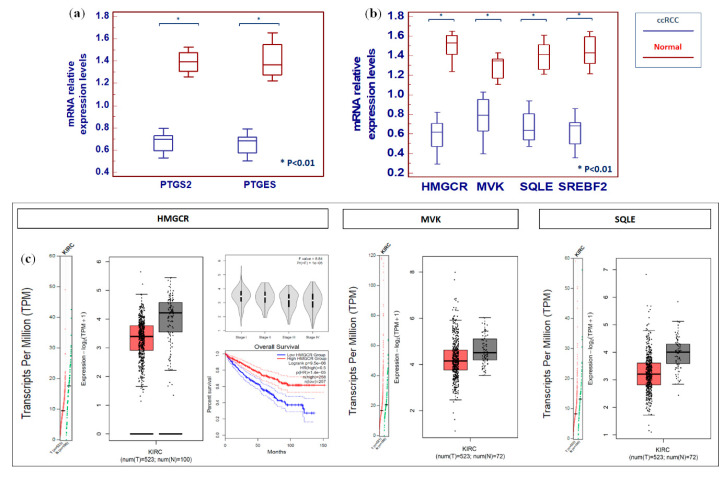
Analysis of gene expression by real-time PCR of prostaglandin-endoperoxide synthase 2 (PTGS2) and prostaglandin E synthase (PTGES) (**a**), and 3-hydroxy-3-methyl-glutaryl-coenzyme A reductase (HMGCR), mevalonate kinase (MVK), squalene epoxidase (SQLE), and sterol regulatory element-binding transcription factor 2 (SREBF2) (**b**). Data mining of The Cancer Genome Atlas (TCGA) clear cell renal cell carcinoma patient cohort (KIRC) using GEPIA2 for HMGCR, MVK, and SQLE genes (**c**).

Functional analysis showed that alterations in arachidonic acid metabolism and prostaglandins production had a great impact on the ccRCC lipidomic profile. Therefore, the expression of cyclooxygenase 2/prostaglandin-endoperoxide synthase 2 (COX2/PTGS2) and prostaglandin E synthase (PTGES) was investigated. Both the transcripts of these genes were reduced in cancer tissue (*p* < 0.01), in accordance with the reduced levels of arachidonic acid derivatives (Figure 10a). Moreover, since cholesterol metabolism-related intermediates were altered in ccRCC, we evaluated the expression of four genes involved in its biosynthesis, namely, 3-hydroxy-3-methyl-glutaryl-coenzyme A reductase (HMGCR), mevalonate kinase (MVK), squalene epoxidase (SQLE), and sterol regulatory element-binding transcription factor 2 (SREBF2). Interestingly, all these genes were downregulated in ccRCC, in accordance with the reduced levels of tissue cholesterol content (Figure 10b). These findings were confirmed by data mining of the cancer genome atlas (TCGA), clear cell renal cell carcinoma patient cohort (KIRC), using GEPIA2 (Figure 10c) [23].

**Figure 11:**
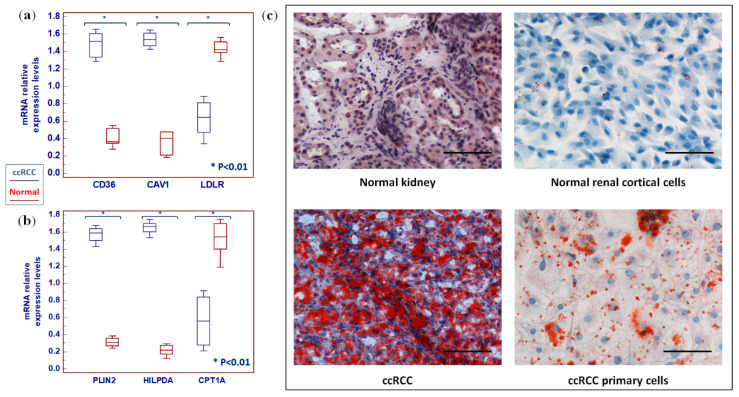
Analysis of gene expression by real time PCR of CD36, caveolin 1 (CAV1) and low-density lipoprotein receptor (LDLR) (**a**), and perilipin 2 (PLIN2), hypoxia inducible lipid droplet-associated (HILPDA), and carnitine palmitoyltransferase 1A (CPT1A) (**b**). Representative images of normal and neoplastic kidney tissue (ccRCC) and normal cortical and ccRCC primary cell cultures captured after Oil Red O (ORO) staining at original magnification of 200×. Scale bars = 100 µm (**c**).

Next, we evaluated the lipid and cholesterol receptors, and found increases in CD36 and caveolin 1 (CAV1) and a decrease in low density lipoprotein receptor (LDLR) transcript levels in ccRCC compared to normal tissue (Figure 11a). Finally, analysis of lipid storage markers showed an increased expression of perilipin 2 (PLIN2) and hypoxia inducible lipid droplet-associated (HILPDA), and reduced levels of carnitine palmitoyltransferase 1A (CPT1A) mRNA in tumor specimens compared to normal kidney (Figure 11b). Consistent with these findings, tumor tissue and primary renal cancer cells showed an increased level of lipid storage, as assessed by Oil red O (ORO) staining (Figure 11c). 

The results of transcriptomic and lipidomic analyses were confirmed by data mining of the Oncomine microarray gene expression datasets (Figure S1) and the Gene Expression Profiling Interactive Analysis 2 (GEPIA2) database, and by using the Metabologram data portal [23,24,25].

Finally, large-scale genomic studies performed in sporadic ccRCC, identified significantly mutated genes including VHL, PBRM1, SETD2, and BAP1. Spearman correlation analysis between these genes and ACLY, SREBF1, SCD1, and ELOVLs in the The Cancer Genome Atlas - clear cell renal cell carcinoma (TGCA-KIRC) patient cohort was shown in Table S1.

### 2.5. Preoperative Serum Total Cholesterol Is an Independent Prognostic Factor for Patients with ccRCC

**Figure 12:**
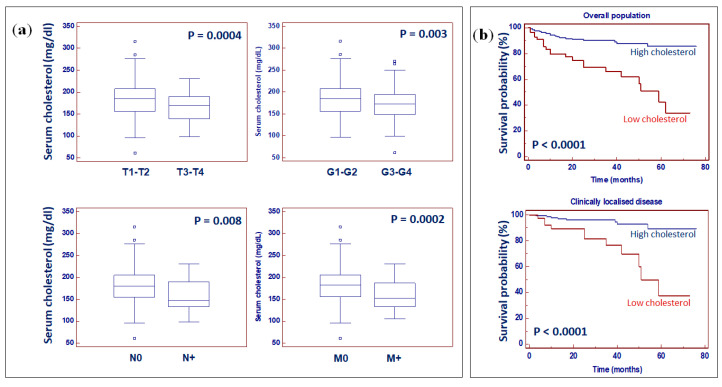
Median levels of serum cholesterol in ccRCC patients stratified according to pathological stage, Fuhrman grade, lymph node involvement, and visceral metastases (**a**). Kaplan-Meier cancer-specific survival (CSS) curves, stratified by serum cholesterol levels in the overall population and in a subset of patients with localized disease (**b**). Small squares indicate outlier in box-and-whisker plots.

Statistically significant differences resulted between serum cholesterol values and clinical stage (*p* = 0.0004), Fuhrman grade (*p* = 0.003), lymph node involvement (*p* = 0.008), and visceral metastases (*p* = 0.0002) (Figure 12a).

To evaluate the association between patients’ survival and the preoperative serum cholesterol, we classified the entire population according to the cut-off provided by receiver operating characteristic (ROC) curve analysis. Detailed clinical and pathological characteristics of the patients are summarized in Table S2.

Kaplan–Meier survival curves for cancer-specific survival (CSS), stratified by preoperative serum cholesterol levels, in the overall population and in a subset of patients with localized disease (pT1-2, N0, M0), are shown in Figure 12b. 

CSS was significantly decreased in patients with low serum levels of total cholesterol. Univariate analysis for the predefined variables showed that the pathological stage, presence of nodal and visceral metastases, Fuhrman grade, presence of necrosis, tumor size, increased body mass index (BMI), and low levels of cholesterol, were significantly associated with the risk of death (Table 1). At multivariate analysis by Cox regression modeling, the pathological stage, presence of nodal and visceral metastases, Fuhrman grade, and reduced circulating levels of total cholesterol were independent adverse prognostic factors for CSS (Table 1).

## 3. Discussion

The prevalence of RCC, as with many other urologic tumors including prostate and bladder cancers, is increased in patients with metabolic disorders [26,27,28,29,30,31]. In particular, obesity is a well-established risk factor for renal cancer, and a recent study has demonstrated that this association appears to be already established during late adolescence [32]. An altered lipid metabolism represents a peculiar hallmark of cancer cells, since lipids play important roles in many aspects of cancer biology such as cell signaling, membrane formation, cellular proliferation and metastatization. 

Our integrated lipidomics-transcriptomics approach revealed that ccRCC tissues exhibit a reprogramming of fatty acid metabolism in association with an altered expression of lipid metabolism-associated genes. In particular, we found a massive change in nearly all the free fatty acid levels with accumulations of many EFAs and LCFAs. These changes, in association with an increased expression of genes involved in fatty acid uptake and/or synthesis (ACLY, CD36 and CAV1), were suggestive of an altered membrane remodeling in ccRCC. Palmitate (16:0) is the main product of de novo lipogenesis and can be elongated and desaturated through the activity of SCD1, and ELOVLs to generate additional SFAs, MUFAs and PUFAs including palmitoleate (16:1n7), stearate (18:0), and oleate (18:1n9). These FAs, in turn, can be used for the synthesis of more complex lipids. The accumulation of PUFAs, in association with reduced levels of lysolipids, sphingolipids and cholesterol, suggested an alteration in cancer cell membrane permeability and fluidity and in phospholipids remodeling (Lands’ cycle), as demonstrated by other studies [33,34,35,36,37].

SCD1 regulation is very complex and its activity and expression is controlled by a large number of effectors including molecular, hormonal and dietary factors [38,39]. SCD1 gene promoter contains binding sites for a variety of transcription factor including SREBF1c, liver X receptor (LXR), peroxisome proliferator-activated receptor alpha (PPARα), nuclear transcription factor Y (NF-Y), specificity protein 1 (SP1), CCAAT/enhancer-binding protein alpha (C/EBP-α), neurofibromin 1 (NF1), and peroxisome proliferator-activated receptor gamma coactivator 1 alpha (PGC1-α) [38]. In addition, the activation of the hypoxia-inducible factor (HIF) pathway, which is essential for ccRCC progression, is another important modulator of SCD1 expression in cancer cells. Zhang et al. showed that SCD1 was upregulated in ccRCC cell lines under hypoxia, and that HIF-2α and SCD1 had synergistic effects in sustaining cancer cell survival and migration [40]. These effects were due to a positive feedback loop between HIF-2α and SCD1, mediated by PI3K/Akt pathway activation [40]. 

A previous study showed that increased SCD1 expression supported ccRCC viability, and when SCD1 was blocked using the small molecule inhibitor A939572, a significantly reduced cancer cell proliferation and the induction of apoptosis were observed [41]. Gene expression profile analysis showed that the loss of SCD1 activity in ccRCC cells induced an increased expression of endoplasmic reticulum (ER) stress genes associated with the unfolded protein response (UPR) [41]. A growing body of literature has demonstrated that UPR activation alters the chemosensitivity of cancer cells, and in particular it has been shown that the ER stress response sensitizes various solid tumor cells to cisplatin-induced apoptotic death [42]. RCC is a typical chemo-resistant tumor, and despite the introduction of novel targeted therapies, no evidence of complete responses has been reported [43,44]. On the basis of these data, we explored the role of SCD1 in sustaining chemotherapy resistance in ccRCC. Interestingly, inhibition of SCD1 activity reduced cell viability and sensitized cancer cells to cisplatin-induced apoptotic death.

On this basis, pharmacological targeting of SCD1 represents an attractive approach, especially in combination with other target therapies approved for metastatic tumors [41,43,44]. For example, the inhibition of SCD1 activity potentiates the inhibitory effects of gefitinib and temsirolimus on cancer cell proliferation in non-small cell lung cancer and ccRCC, respectively [41,45]. Interestingly, the treatment of cancer cells with SCD1 inhibitors can trigger the AMPK-dependent autophagic pathway, providing a resistance mechanism against SCD1 inhibition [46]. In this scenario, therapeutic strategies that target SCD1 may be potentiated by combination with molecules that target the autophagic machinery [47].

Cholesterol metabolism is often reprogrammed in cancer cells [48]. Our findings demonstrated that the reduced accumulation of cholesterol metabolism-related intermediates in renal cancer tissue was associated with downregulation of the low-density lipoprotein receptor (LDLR) gene and correlated with the serum total cholesterol levels. Moreover, we found that preoperative serum cholesterol was an independent prognostic factor for CSS, in a cohort of 450 patients who underwent radical or partial nephrectomy for ccRCC. As serum cholesterol is routinely assessed in ccRCC patients when they are admitted to hospital or during follow-up after surgery, its use in clinical practice may provide a useful tool for risk stratification.

Many studies have demonstrated that tumor-associated inflammation has an important role in tumorigenesis, cancer progression, and metastatization [49]. In this scenario, it has been shown that eicosanoids, including prostaglandins, play a critical role in these processes [50]. Clear cell RCC is characterized by the activation of extrinsic and intrinsic inflammatory pathways and many studies have reported a correlation between clinical outcomes and laboratory markers of systemic inflammation [51,52]. Paradoxically, we found a reduced accumulation of arachidonic acid-derived metabolites, including prostaglandin 2, as well as a reduced expression of COX2/PTGS2 and PTGES transcripts in cancer tissue. These findings suggest that the regulation of cancer-related inflammation in ccRCC is quite complex and may involve other mechanisms such as adaptive immune responses and the complement system [53].

A feature of ccRCC is the presence of intracellular lipid droplets (LDs), which have the function of releasing lipid species for membrane biosynthesis and sustaining endoplasmic reticulum homeostasis. Recently, it has been shown that two LD-associated proteins (PLIN2 and HILPDA) are overexpressed in ccRCC, regulate lipid storage and enrich lipids that contain polyunsaturated fatty acyl side chains [54]. Moreover, PLIN2 is required for ER homeostasis and cell viability in ccRCC cell lines and xenograft tumors, and its depletion triggers the UPR, cell cycle withdrawal, and cell death [54]. In addition, the mechanism of lipid deposition is favored by repression of carnitine palmitoyltransferase 1A (CPT1A), an enzymatic component of mitochondrial FA transport. In ccRCC, hypoxia inducible factors (HIFs) are responsible for inhibiting CPT1A expression, reducing FA transport into mitochondria, and rerouting FA to LDs for storage [55]. In accordance with these findings, we found an increased expression of PLIN2 and HILPDA, and reduced levels of CPT1A transcripts in cancer tissue. Moreover, ORO staining confirmed the increased lipid storage in cancer cells.

## 4. Materials and Methods

### 4.1. Study Population and Tissue Collection

Primary renal tumor (*n* = 20) and paired non-neoplastic samples (*n* = 20) were retrieved from patients with ccRCC. Patients with eGFR < 60 mL/min/1.73 m^2^ and metabolic diseases (including diabetes mellitus) were excluded from the study. Informed consent was obtained from all individual participants included in the study. All procedures were in accordance with the ethical standards of the institutional and/or national research committee and with the 1964 Helsinki Declaration and its later amendments or comparable ethical standards. The research project was approved by the local Ethics Committee (n. 143/CE/2015).

### 4.2. Metabolite Analysis

#### 4.2.1. Sample Preparation

Metabolic analyses were performed at Metabolon Inc. All tissue samples were stored at −80 °C. The automated MicroLab STAR^®^ system (Hamilton, Reno, NV, USA) was used for sample preparation. Recovery standards were used in the extraction process for quality control (QC). A proprietary series of organic and aqueous extractions were used for sample preparation. The resulting extract was split into two aliquots: one for liquid chromatography (LC) and one for gas chromatography (GC) analysis. Samples were placed on a TurboVap^®^ (Zymark, Hopkinton, MA, USA) for the organic solvent removal and then frozen and dried under vacuum. Tables S3 and S4 list the QC compounds. 

#### 4.2.2. Liquid Chromatography/Mass Spectrometry (LC/MS, LC/MS)

The LC/MS platform was composed by a Waters ACQUITY UPLC (Waters Corporation, Milford, CT, USA) and a Thermo-Finnigan LTQ MS (Thermo Fisher, Waltham, MA, USA). The sample extract was divided into two parts, dried, and rehydrated in acidic or basic LC-compatible solvents. The analysis was performed using acidic positive ion and basic negative ion optimized conditions using different dedicated columns. The extracts, rehydrated in acidic conditions, were eluted using water/methanol with 0.1% Formic acid. For the basic extracts, the reconstitution was performed using water and methanol, with 6.5mM Ammonium Bicarbonate.

#### 4.2.3. Gas Chromatography/Mass Spectrometry (GC/MS) 

GC/MS analysis was performed using sampled re-dried under vacuum desiccation for a minimum of 24 h and then derivatized using bistrimethyl-silyl-trifluoroacetamide (BSTFA) (Sigma Aldritch, Saint Louis, MO, USA). The GC column was 5% phenyl and the temperature conditions ranged from 40 °C to 300 °C. Samples were analyzed using a Thermo-Finnigan Trace DSQ fast-scanning single-quadrupole MS (Thermo Fisher, Waltham, MA, USA).

#### 4.2.4. Accurate Mass Determination and MS/MS Fragmentation (LC/MS), (LC/MS/MS) 

The LC/MS part of the platform consists of a Waters ACQUITY UPLC and a Thermo-Finnigan LTQ-FT MS. Fragmentation spectra (MS/MS) were obtained in a data-dependent manner. If needed, targeted MS/MS was used.

#### 4.2.5. Compound Identification

Compounds identification was performed by comparison to library entries of purified standards or recurrent unknown entities.

### 4.3. Bioinformatics and Statistical Analyses

MedCalc 9.2.0.1 (MedCalc software, Mariakerke, Belgium) and “R” (http://cran.r-project.org) [56] were used for statistical analyses. Comparisons of median values between groups were performed using Mann-Whitney U or Kruskal-Wallis test, as appropriate. Spearman’s test was used to study the correlation between serum and tissue cholesterol levels.

Cancer-specific survival (CSS) was estimated by using the Kaplan–Meier method and compared with the log-rank test. The Cox proportional hazards regression model was for univariable and multivariable analyses. *p*-values below 0.05 were considered statistically significant.

### 4.4. Integration of Metabolomic and Transcriptomic Data

Exon array analysis of 20 total samples (10 ccRCC neoplastic tissues and paired non-neoplastic samples) was performed (GEO accession number: GSE47032).

Metabolite set enrichment (MSEA) and transcriptomics–metabolomics data integration was performed using MetaboAnalyst 4.0 (https://www.metaboanalyst.ca) [20]. Gene set enrichment analysis (GSEA) [22], performed across renal cancer datasets, revealed which pathways were enriched in ccRCC. The normalized enrichment score (NES) gave an estimate of the importance and direction of pathway enrichment.

ChemRICH (https://chemrich.fiehnlab.ucdavis.edu) was used for biochemical pathway enrichment analysis [21].

### 4.5. Real Time Polymerase Chain Reaction (PCR)

Total RNA was reverse transcribed using the High-Capacity cDNA Reverse Transcription Kit (Applied Biosystems Foster City, CA, USA), according to the manufacturer’s instructions. Quantitative real-time PCR was performed using the iQTM SYBR Green Supermix buffer (6mMMgCl2, dNTPs, iTaq DNA polymerase, SYBR Green I, fluorescein and stabilizers) (BIO-RAD Laboratories, Hercules, CA, USA). The primers used in this study are reported in Table S5.

MiniOpticon Real-Time PCR detection system (BIO-RAD Laboratories, Hercules, CA, USA) was used for mRNA levels quantification. The following conditions were used: polymerase activation at 95 °C for 3 min, followed by 45 cycles at 95 °C for 10 s, 60 °C for 30 s. Expression was determined using the 2^-ΔΔCt^ method used for quantification. β-Actin was used for normalization.

### 4.6. Data Mining Using the Oncomine Gene Expression Microarray Datasets, Gene Expression Profiling Interactive Analysis 2 (GEPIA2) Database, and Metabologram Data Portal

The Oncomine database (https://www.oncomine.org/resource/login.html) [24] was explored for publicly available transcriptomics data analysis.

The GEPIA2 database (http://http://gepia2.cancer-pku.cn) [23] was also used to validate the expression of genes involved in ccRCC lipid metabolism. In this web-based resource, the data from the Cancer Genome Atlas (TGCA) and Genotype-Tissue Expression (GTEx) are available for validation analysis. The clear cell renal cell carcinoma (KIRC) cohort, includes 523 ccRCC and 100 normal kidney specimens.

In addition, the metabolic pathways were explored using the Metabologram data portal (http://sanderlab.org/kidneyMetabProject) [25], a web-based application that combines data derived from TCGA database and MSKCC metabolomics dataset.

### 4.7. Primary Cell Cultures from Renal Tissues

Primary tumor and normal cell cultures were obtained from tumor (ccRCC) and normal kidney tissue specimens as previously described [57]. Immunocytochemistry by using EpCAM and CA IX was performed for cell characterization.

### 4.8. Cell Viability Assay

Cell viability after exposure to 75 nmol/L of A939572 or to A939572 and 10 μM cis-Diamminedichloroplatinum (II) (cisplatin) was evaluated using MTT assay as previously described [38]. In the first part of the experiment, the cells were exposed to A939572 for 72h or incubated in medium alone. In the second part of the experiment, after 24 h the cells were treated with cisplatin 10 μM for 1 h and 2 h. Each experiment was performed in triplicate.

### 4.9. Oil Red O Staining

Oil Red O (ORO) staining was performed on formalin-fixed primary cells and tissue samples, as previously described [19,58].

### 4.10. Availability of Data and Material

The datasets generated and/or analyzed during the current study are available in the GEO repository:

1. Accession number GSE47032;
2. Accession number GSE15641;
3. Accession number GSE41485.

## 5. Conclusions

In conclusion, our study showed that ccRCC is characterized by lipid metabolism reprogramming associated with a switch in adipogenic gene signatures. The accumulation of very long-chain FAs and PUFAs is sustained by overexpression of SCD1 and ELOVLs, and the inhibition of SCD1 activity decreases cell viability and improves cisplatin susceptibility, suggesting that this pathway can regulate chemotherapy resistance in ccRCC.

## Figures and Tables

**Table 1:** Univariate and multivariate analyses for cancer-specific survival.

Variable	Category	Univariate		Multivariate	
		HR (95% CI)	*p*-Value	HR (95% CI)	*p*-Value
T stage	T3/4 vs. T1/2	4.44 (2.38–8.27)	0.0001	1.91 (1.18–3.09)	0.002
N stage	N+ vs. N0	5.49 (2.53–11.91)	0.0001	2.48 (1.41–3.96)	0.001
M stage	M+ vs. M0	7.62 (4.07–14.25)	0.0001	3.81 (1.89–7.68)	0.0002
Grade	G3/4 vs. G1/2	5.37 (2.86–10.09)	0.0001	3.35 (1.67–6.72)	0.001
Necrosis	Yes vs. No	3.47 (2.11–5.91)	0.0001	-	-
Tumor size	Continuous	1.13 (1.07–1.21)	0.0001	-	-
BMI	Continuous	0.86 (0.79–0.94)	0.01		
Total serum cholesterol	≤155 vs. >155 mg/dL	2.21 (1.96–3.81)	<0.001	1.72 (1.43–2.51)	0.001

## Supplementary Materials

The following are available online at https://www.mdpi.com/2218-1989/10/12/509/s1, Figure S1: Oncomine analysis, Table S1: Spearman’s rank correlation coefficients between genes using TCGA clear cell renal cell carcinoma patient cohort (KIRC), Table S2: Clinical and pathological characteristics of patients who underwent radical or partial nephrectomy for ccRCC, Table S3: Description of Metabolon QC Samples, Table S4: Metabolon QC Standards, Table S5: Primers used for real time PCR.
