# Supplementary material for: Integration of Lipidomics and Transcriptomics Reveals Reprogramming of the Lipid Metabolism and Composition in Clear Cell Renal Cell Carcinoma
Source: Metabolites. 2020 Dec 13;10(12):509. doi: 10.3390/metabo10120509 (PMC7763669; doi:10.3390/metabo10120509)
Supplement: Supplementary file 1 [file metabolites-10-00509-s001.zip › supplementary files/Table S5.docx]

| ACLY | F: 5’-AAGTGGGGTGACATCGAGTT-3’ |
| --- | --- |
|  | R: 5’-CTTTGGGGTTCAGCAAGGTC-3’ |
| SCD1 | F: 5′-GTCCTTATGACAAGAACATTAGCC-3′  R: 5′-AATCAATGAAGAATGTGGTGAAG-3’ |
| ELOVL2 | F: 5’-TGTGTTTCCATCTATGCACAAGT-3’  R: 5’- CAGCTGAGCCTGTGTGAGAT-3’ |
| ELOVL5 | F: 5’-TTCATCCTGCGCAAGAACAACCAC-3’  R: 5’-ATGGAAGGGACTGACGACAAACCA-3’ |
| SREBF1 | F: 5’- TTGCCGACCCTGGTGAGT -3’ |
|  | R: 5’- AATGGCGTTGTGGGCTGT -3’ |
| SREBF2 | F: 5’-AACGGTCATTCACCCAGGTC-3’ |
|  | R: 5’-GGCTGAAGAATAGGAGTTGCC-3’ |
| HMGCR | F: 5′-TGATTGACCTTTCCAGAGCAAG-3′ |
|  | R: 5′-CTAAAATTGCCATTCCACGAGC-3′ |
| MVK | F: 5′-CATGGCAAGGTAGCACTGG-3′ |
|  | R: 5′-GATACCAATGTTGGGTAAGCTGA-3′ |
| SQLE | F: 5′-TGGTTACATGATTCATGATC-3′ |
|  | R: 5′-TACTGAACTCCCATCACAAC-3′ |
| PTGS2 | F: 5’-ACCGCAAACGCTTTATGCTG-3’ |
|  | R: 5’-AAAGATGGCATCTGGCCGA-3’ |
| PTGES | F: 5’- CTGGTCATCAAGATGTACGTG-3’ |
|  | F: 5’- GGGTAGATGGTCTCCATGTC-3’ |
| CD36 | F: 5′-TCACTGCGACATGATTAATGGTAC-3 |
|  | R: 5′-ACGTCGGATTCAAATACAGCATAGAT-3′ |
| CAV1 | F: 5’-TTCTGGGCTTCATCTGGCAAC-3’ |
|  | R: 5’-GCTCAGCCCTATTGGTCCACTTTA-3’ |
| LDLR | F: 5’-GGATCCGTTCATGGCTTCA-3’ |
|  | R: 5’-ATTGGGCCACTGAATGTTTT-3’ |
| PLIN2 | F: 5’-CATTGTGTTGCCTCTGTTAC-3’ |
|  | R: 5’-CCTTTTCAGATCACACCAGA-3’ |
| HILPDA | F: 5’-AAGCATGTGTTGAACCTCTACC-3’  R: 5’-TGTGTTGGCTAGTTGGCTTCT-3’ |
| CPT1A | F: 5’- TGGATCTGCTGTATATCCTTC-3’  R: 5’-AATTGGTTTGATTTCCTCCC-3’ |
| B-ACTIN | F: 5’-AATCTGGCACCACACCTTCT-3’ |
|  | R: 5’-AGCCTGGATAGCAACGTACA-3’ |

**Table S5.** Primers used for real time PCR**.**
