# Supplementary material for: Integration of Lipidomics and Transcriptomics Reveals Reprogramming of the Lipid Metabolism and Composition in Clear Cell Renal Cell Carcinoma
Source: Metabolites. 2020 Dec 13;10(12):509. doi: 10.3390/metabo10120509 (PMC7763669; doi:10.3390/metabo10120509)
Supplement: Supplementary file 1 [file metabolites-10-00509-s001.zip › supplementary files/Table S4.docx]

| Type | Description | Purpose |
| --- | --- | --- |
| DS | Derivatization Standard | Assess variability of derivatization for GC/MS samples. |
| IS | Internal Standard | Assess variability and performance of instrument. |
| RS | Recovery Standard | Assess variability and verify performance of extraction and instrumentation. |

**Table S4.** Metabolon QC Standards.
