# Supplementary material for: Integration of Lipidomics and Transcriptomics Reveals Reprogramming of the Lipid Metabolism and Composition in Clear Cell Renal Cell Carcinoma
Source: Metabolites. 2020 Dec 13;10(12):509. doi: 10.3390/metabo10120509 (PMC7763669; doi:10.3390/metabo10120509)
Supplement: Supplementary file 1 [file metabolites-10-00509-s001.zip › supplementary files/Table S3.docx]

| Type | Description | Purpose |
| --- | --- | --- |
| MTRX | Large pool of human plasma maintained by Metabolon that has been characterized extensively. | Assure that all aspects of Metabolon process are operating within specifications. |
| CMTRX | Pool created by taking a small aliquot from every customer sample. | Assess the effect of a non-plasma matrix on the Metabolon process and distinguish biological variability from process variability. |
| PRCS | Aliquot of ultra-pure water | Process Blank used to assess the contribution to compound signals from the process. |
| SOLV | Aliquot of solvents used in extraction. | Solvent blank used to segregate contamination sources in the extraction. |

**Table S3.** Description of Metabolon QC Samples.
