# Supplementary material for: Integration of Lipidomics and Transcriptomics Reveals Reprogramming of the Lipid Metabolism and Composition in Clear Cell Renal Cell Carcinoma
Source: Metabolites. 2020 Dec 13;10(12):509. doi: 10.3390/metabo10120509 (PMC7763669; doi:10.3390/metabo10120509)
Supplement: Supplementary file 1 [file metabolites-10-00509-s001.zip › supplementary files/Table S2.docx]

| **Variable** | **n=450** |
| --- | --- |
| Age (years)  median  range | 62  26-88 |
| Gender  Male  Female | 291 (64.7%)  159 (35.3%) |
| Dimensions (cm)  median  range | 4.5  0.5 -22 |
| Pathological stage  pT1  pT2  pT3  pT4 | 313 (69.5%)  49 (10.8%)  83 (18.5%)  5 (1.2%) |
| pN+ | 34 (7.6%) |
| cM+ | 37 (8.3%) |
| Fuhrman grade  G1-2  G3-4 | 324 (72%)  126 (28%) |

**Table S2.** Clinical and pathological characteristics of patients who underwent radical or partial nephrectomy for ccRCC.
