# Supplementary material for: Integration of Lipidomics and Transcriptomics Reveals Reprogramming of the Lipid Metabolism and Composition in Clear Cell Renal Cell Carcinoma
Source: Metabolites. 2020 Dec 13;10(12):509. doi: 10.3390/metabo10120509 (PMC7763669; doi:10.3390/metabo10120509)
Supplement: Supplementary file 1 [file metabolites-10-00509-s001.zip › supplementary files/Table S1.docx]

|  | VHL | PBRM1 | SETD2 | BAP1 |
| --- | --- | --- | --- | --- |
| SCD1 | 0.12 | 0.19 | **NS** | -0.15 |
| ELOVL2 | 0.18 | 0.29 | 0.17 | 0.14 |
| ELOVL5 | 0.52 | 0.63 | 0.5 | 0.16 |
| ACLY | 0.32 | 0.53 | 0.49 | 0.08 |
| SREBF1 | 0.27 | 0.17 | 0.26 | 0.27 |

**Table S1**: Spearman's rank correlation coefficients between genes using TCGA clear cell renal cell carcinoma patient cohort (KIRC). NS: not significant
