# Supplementary material for: Integration of Lipidomics and Transcriptomics Reveals Reprogramming of the Lipid Metabolism and Composition in Clear Cell Renal Cell Carcinoma
Source: Metabolites. 2020 Dec 13;10(12):509. doi: 10.3390/metabo10120509 (PMC7763669; doi:10.3390/metabo10120509)
Supplement: Supplementary file 1 [file metabolites-10-00509-s001.zip › supplementary files/Figure S1.docx]

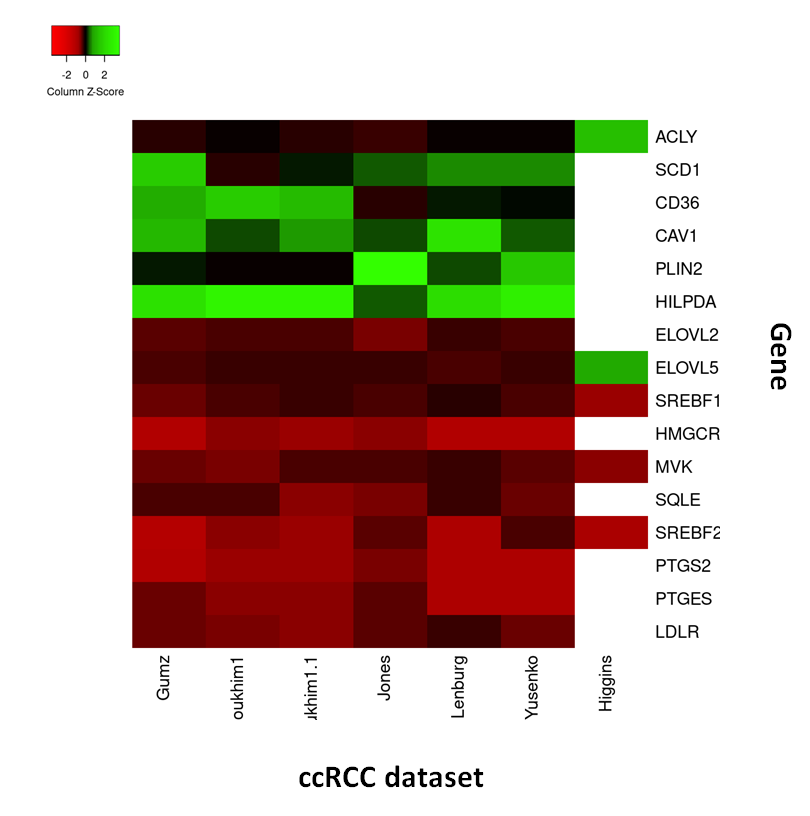


**Figure S1.** Oncomine analysis. Oncomine heatmap analysis in 7 published ccRCC datasets for expression of genes evaluated in the study.
